# P-831. Six Years Retrospective Study on *Staphylococcus aureus* Bacteremia: Impact of Persistance and COVID-19 Pandemic on 30-Days Mortality

**DOI:** 10.1093/ofid/ofae631.1023

**Published:** 2025-01-29

**Authors:** Francesco Molà, Lidia Gazzola, Ottavia Viganò, Roberto Castoldi, Francesca Di Bartolomeo, Giorgio Giannetta, Francesca Bai, Giulia Marchetti

**Affiliations:** ASST-Santi Paolo e Carlo, Department of Health Science, University of Milan, Milan, Milano, Lombardia, Italy; ASST-Santi Paolo e Carlo, Department of Health Science, University of Milan, Milan, Milan0, Lombardia, Italy; ASST-Santi Paolo e Carlo, Department of Health Science, University of Milan, Milan, Milan0, Lombardia, Italy; ASST-Santi Paolo e Carlo, Department of Health Science, University of Milan, Milan, Milan0, Lombardia, Italy; ASST-Santi Paolo e Carlo, Department of Health Science, University of Milan, Milan, Milan0, Lombardia, Italy; ASST-Santi Paolo e Carlo, Department of Health Science, University of Milan, Milan, Milan0, Lombardia, Italy; ASST-Santi Paolo e Carlo, Department of Health Science, University of Milan, Milan, Milan0, Lombardia, Italy; Clinic of Infectious Diseases, Department of Health Sciences, ASST Santi Paolo e Carlo, University of Milan, Milan, Italy, Milano, Lombardia, Italy

## Abstract

**Background:**

*S. aureus* bacteremia (SAB) is characterized by high risk of complications and a high mortality. Persistent *S. aureus* bacteremia (P-SAB) has been associated with worse outcome. The COVID-19 pandemic might have further increased the mortality for this condition. Our study aims to assess the prevalence and risk factors for P-SAB and its 30-day all-cause mortality and relative risk factors, with particular attention to the possible influence of SARS-CoV-2 infection.

**Table 1.**
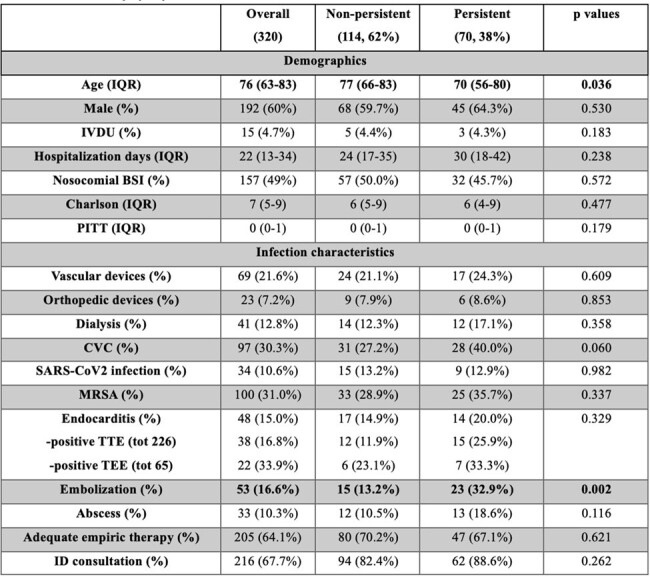
Study population characteristics Quantitative data are presented as median, interquartile range, qualitative data as absolute numbers, percentages; p values for comparison between persistent and non-persistent SAB by Mann-Whitney test and Chi-square test for continuous and categorical variables, respectively.

(IQR: interquartile range; IVDU: intravenous drug users; BSI: bloodstream infection; CVC: central venous catheter; MRSA: methicillin-resistant S. aureus; TTE: transthoracic echocardiography; TEE: transesophageal echocardiography; ID: infectious diseases).

**Methods:**

This is a retrospective study including all adult patients with monomicrobic SAB, from January 2017 to December 2022 at San Paolo Hospital, Milan. P-SAB was defined as a positive blood culture at 72 hours from the beginning of an active antibiotic therapy. Patients who didn’t perform follow-up blood cultures were excluded for analysis evaluating risk factors and mortality for P-SAB.

Risk factors for P-SAB were evaluated by univariable logistic regression analysis. Kaplan-Meier curves with log-rank test and multivariable Cox regression analysis were performed for all-cause 30-day mortality, persistance and mortality risk factors.

**Table 2.**
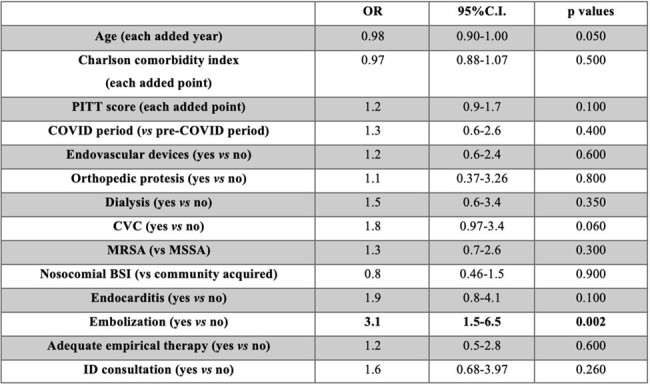
Univariable analysis for risk factors associated with persistent S. aureus bloodstream infections. (OR: odds ratio; C.I.: confidence interval; CVC: central venous catheter; MRSA: methicillin-resistant S. aureus; MSSA: methicillin-sensible S. aureus; BSI: bloodstream infection; ID: infectious disease)

**Results:**

In total 320 patients were diagnosed with SAB during the study period (population characteristics in Table 1); 184 patients had available follow-up blood cultures, 70 (38%) presented a P-SAB. Embolization was a risk factor for persistent bacteremia (OR 3.1, 95%C.I. 1.5-6.5, p=0.002), as well as the presence of CVC at SAB onset (OR 1.8, 95%C.I. 0.97-3.4, p=0.060) (Table 2). All-cause 30-day mortality for SAB was 25.3% (95%C.I. 19.7-30.9%), survival curve found no differences in mortality (Log-rank=0.080) for P-SAB (Figure 1). At multivariable analysis P-SAB presented more risk of mortality (aHR 2.06, 95% C.I. 0.86-4.9, p=0.100), along with a concomitant SARS-CoV-2 infection (HR 2.48, 95% C.I. 1.37-4.48, p=0.002) and comorbidities (aHR 1.16, 95% C.I. 1.03-1.32, p=0.020) (Table 3).

**Figure 1.**
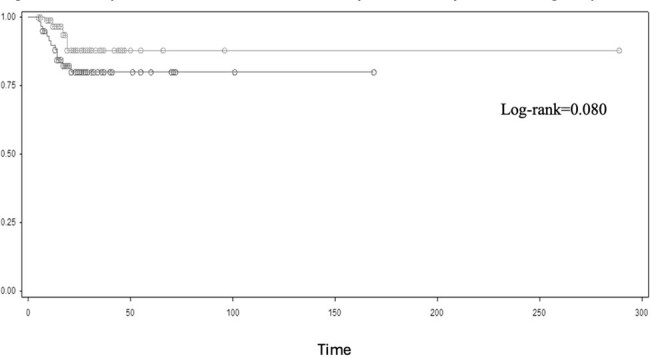
Kaplan-Meier curve for 30-days mortality according to persistent SAB. (Grey curve: non-persistent S. aureus bacteremia; Black curve: persistent S. aureus bacteremia. P value by log-rank test)

**Conclusion:**

P-SAB is associated with the presence of endovascular catheters and septic embolization, determining a higher 30-day mortality compared to non-P-SAB. Moreover SARS-CoV-2 coinfection showed to double mortality risk for SAB. Optimal management of patients with P-SAB must be further elucidated to reduce their relevant impact in daily practice.

**Table 3.**
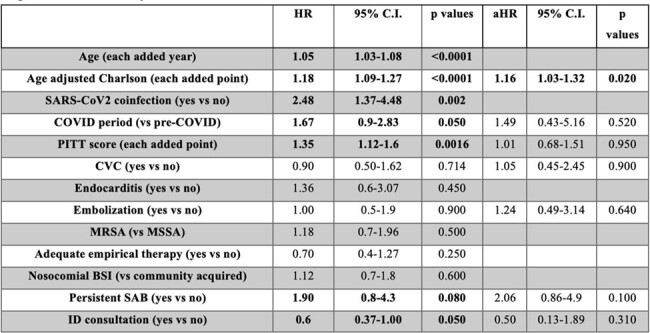
Factors associated with 30-day mortality by fitting a univariable and multivariable Cox-regression analysis. (HR: hazard ratio; C.I.: confidence interval; aHR, adjusted hazard ratio; CVC: central venous catheter; MRSA: methicillin-resistant S. aureus; BSI: bloodstream infection; SAB: S. aureus bloodstream infection; ID: infectious diseases).

**Disclosures:**

**All Authors**: No reported disclosures

